# Development and validation of a video-based intervention on self-care practices for patients with hypertension in Malaysian primary care settings

**DOI:** 10.51866/oa.480

**Published:** 2024-06-25

**Authors:** Raja Ibrahim Raja-Ismail, Siti Fatimah Badlishah-Sham, Nik Munirah Nik-Nasir, Mohamad Rodi Isa

**Affiliations:** 1 MBBCh BAO, MMed Fam Med, Department of Primary Care Medicine, Faculty of Medicine, Universiti Teknologi MARA, Sg Buloh Campus, Jalan Hospital, Sungai Buloh, Selangor, Malaysia. Email: sfatimah31@uitm.edu.my; 2 MBBS, Department of Primary Care Medicine, Faculty of Medicine Universiti Teknologi MARA, Sg Buloh Campus, Jalan Hospital, Sungai Buloh, Selangor, Malaysia.; 3 BPharm, MClin Pharm, MBChB, MMed Fam Med, Department of Primary Care Medicine, Faculty of Medicine, Universiti Teknologi MARA, Sg Buloh Campus, Jalan Hospital, Sungai Buloh, Selangor, Malaysia.; 4 BPharm, MClin Pharm, MBChB, MMed Fam Med, Department of Primary Care Medicine, Faculty of Medicine, Universiti Teknologi MARA, Sg Buloh Campus, Jalan Hospital, Sungai Buloh, Selangor, Malaysia.

**Keywords:** Hypertension, Self-care practices, Video-based intervention, Validity

## Abstract

**Introduction:**

Self-care practices among patients with hypertension have been shown to improve blood pressure control. Video-based interventions (VBIs) are helpful in enhancing patients’ selfcare practices. However, validated VBIs in the Malay language for patients in primary care settings are scarce. This study aimed to develop and validate a VBI series in the Malay language to educate patients with hypertension on self-care practices in primary care settings.

**Methods:**

This study was conducted in three phases: (1) pre-production, (2) production and (3) post-production. The pre-production phase involved designing the storyboard and scripts, which underwent content validation by content experts and subsequently by patients with hypertension. Once the storyboards and scripts achieved acceptable consensus, the videos were recorded (production phase). The post-production phase included video editing and face validation among patients with hypertension. Statistical analysis included the calculation of the item-level content validation index (I-CVI) and item-level face validation index (I-FVI) during content and face validation, respectively.

**Results:**

The storyboards and scripts for five videos were developed. The I-CVI of all videos was 1.0 after two rounds of content validation among six content experts. The I-CVI of all videos was 1.0 among five patients with hypertension. Five videos were recorded and edited, achieving an I-FVI of 1.0 during face validation among 10 patients.

**Conclusion:**

A VBI series consisting of five videos was developed and validated for use among patients with hypertension in primary care settings to improve their knowledge of self-care practices.

## Introduction

Hypertension is one of the leading causes of morbidity and mortality worldwide and is one of the commonest non-communicable diseases encountered by primary care physicians.^1^ In the Malaysian National Health and Morbidity Survey 2019,^2^ the prevalence of hypertension among adults aged ≥18 years was 30.0% (95% confidence interval=28.57, 31.50). However, only 45% of these adults had their blood pressure controlled.

Self-care practices can be defined as actions directed towards oneself or the environment to regulate one’s functioning in the interest of one’s life, including integrated functioning and well-being.^3^ They have been shown to effectively control blood pressure.^4^ The seven components of self-care practices for hypertension include the Dietary Approach to Stop Hypertension (DASH) diet, weight reduction, physical activity, alcohol reduction, smoking cessation, self-monitoring and medication adherence. However, evidence has shown that there is a low level of self-care practices among patients with hypertension compared to recommendations in hypertension management protocols.^5^

Based on the chronic care model, selfmanagement support tools such as educational sessions, pamphlets, booklets and videos may improve self-care practices.^6^ Video-based interventions (VBIs) are valuable educational tools for patients that have been shown to enhance patients’ self-care practices.^7^ VBIs have also been found to be more effective than printed pamphlets for both short-term behavioural change and longterm retention.^8^ Other advantages of VBIs include cost-effectiveness, standardisation of teaching^9^ and the ability for patients to view the material multiple times at their convenience.^10^

To our knowledge, there is a lack of available VBIs in the Malay language that have been developed and validated to address self-care practices among patients with hypertension in primary care settings. Accordingly, this study aimed to develop and validate a VBI series in the Malay language to educate patients with hypertension on self-care practices based on the information, motivation and behaviour (IMB) theory by Fisher.^11^

## Methods

This study was conducted at a university primary care clinic from July 2022 to August 2023. It was conducted in three phases based on established literature^7^: pre-production phase (Phase 1), production phase (Phase 2) and post-production phase (Phase 3).

### Phase 1: Pre-production

The objective of Phase 1 was to allow the research team to make fundamental decisions regarding the themes, content and design of the VBI series. Theoretical considerations were made based on the IMB theory,^11^ which postulates that providing patients with the correct information and motivation can help them develop self-efficacy to apply self-care practices to achieve optimal blood pressure control.

Based on the IMB theory, the research team, which consisted of two family medicine specialists (FMSs) and one family medicine trainee, outlined the relevant themes and content related to hypertension self-care practices. The main guidelines used as reference included the Clinical Practice Guidelines on the Management of Hypertension (5th edition),^12^ Healthy Plate Guidelines by the Ministry of Health Malaysia,^13^ Malaysian Physical Activity Guidelines (Ministry of Health Malaysia)^14^ and Clinical Practice Guidelines on the Treatment of Tobacco Use Disorders.^15^ The five themes derived for the VBI series are shown in Table 1.

**Table 1:** Themes derived for the development of a video-based intervention series to improve self-care practices for patients with hypertension in primary care settings.

Video	Topic/theme	Learning content
**VIDEO 1** *Pemakanan seimbang untukpesakit darah tinggi* (Balanced diet for patients with high blood pressure)	To improve self-care practice through dietary changes	1. Making dietary changes as per the DASH diet recommendations 2. Showing examples of healthy meal options
**VIDEO 2** *Penurunan berat badan dan aktiviti fizikal untuk tekanan darah tinggi* (Weight loss and physical activity for patients with high blood pressure)	To improve self-care practice through physical activity and weight loss	1. Approaching weight loss through diet and physical activity changes
VIDEO 3 *Berhenti merokok dan pengambilan alkohol* (Cessation of smoking and reduction of alcohol consumption)	To improve self-care practice by quitting smoking and reducing alcohol consumption	1. Educating patients on smoking cessation and the importance of reducing alcohol consumption
VIDEO 4 *Cara mengambil bacaan tekanan darah di rumah* (Method of taking a blood pressure reading at home)	To improve self-care practice through self-monitoring of blood pressure	1. Educating patients on the correct technique of using a home blood pressure monitor
VIDEO 5 *Pematuhan kepada ubat untuk tekanan darah tinggi* (Adherence to medication for high blood pressure)	To improve self-care practice through adherence to medication	1. Educating patients on the fundamentals of anti-hypertensive medications 2. Giving advice on medication adherence

DASH, dietary approach to stop hypertension

Storyboard construction and script writing were then undertaken to organise the content and design of each video based on storyboarding, which is a technique that outlines the video content, designs the scenes and arranges audio and video recording angles.^16^ Construction of the storyboard also included the elements of recall as well as understanding and application of the video content. It is important to ensure that patients are able to comprehend, remember and apply the knowledge gained from videos to increase their self-care practices. Five storyboards were designed in the Malay language using simple layperson terms with a combination of visual and audio information. This process followed the features deemed important for informative videos and depiction of the application skills that patients could apply in their daily activities.^17^ An example of a storyboard (Video 4) is shown in Table 2.

**Table 2:** Storyboard of Video 4 (method of taking a blood pressure reading at home).

Segment	Short description of the segment
***Pengenalan*** **- Introduction** *Video 4: Cara mengambil bacaan tekanan darah di rumah* Video 4: Method of taking a blood pressure reading at home	Slides showing the title of the video and disclaimer information
***Memberi salam* – Greetings** 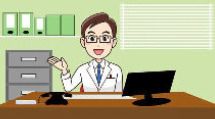	Recording greeting patients and explaining the video contents
***Bahagpan alat mengambil tekanan darah*** **– Sphygmomanometer component** 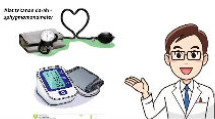	Slides describing the types of sphygmomanometers and their components Explaining how to choose the correct cuff size
***Cara mengambil bacaan tekanan darah* – Technique of taking a blood pressure reading** 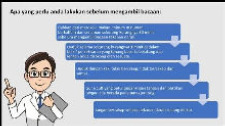	Slides describing the steps needed before taking a blood pressure reading
***Proses mengambil bacaan tekamin darah*** **– Process of blood pressure measurement** 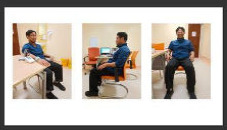	Slides showing the correct position and technique of measuring blood pressure at home

The finalised storyboard and script of the five VBI series were then presented to content experts for content validity. Content validity is known as the amount to which the measuring instrument represents the measured concept.^18^ In this study, an expert panel consisting of six healthcare professionals including two FMSs, a cardiologist, a dietitian, a pharmacist and a physiotherapist was invited to review and score the relevance of the storyboard content and script with regard to self-care practices. The expert panel was well-versed in the management of patients with hypertension and had a minimum of 5 years of working experience in their respective fields. The content validation process was conducted as a face-to-face meeting, in which the experts were shown a PowerPoint presentation of the video storyboards and asked to rate the relevance of the content of the videos.

The content validity was analysed using the item-level content validity index (I-CVI), with the experts scoring 1 (‘not relevant’) to 4 (‘very relevant’) for each video assessed.^18^ Items scoring either 1 or 2 were re-categorised into the ‘not relevant’ category with 0 points each. Items scoring either 3 or 4 were recategorised into the ‘relevant’ category with 1 point each. In general, when there are six or more experts in an expert panel, the I-CVI should not be lower than 0.78.^19^ Herein, the experts were also asked to offer suggestions for improvement of the video content. Based on the I-CVI from the expert panel and their feedback, the storyboards or scripts deemed irrelevant were revised, modified or excluded as appropriate. Once modification was made to the storyboards and scripts, they were then presented again to the expert panel; this process was repeated until a consensus was reached.

The storyboards and scripts then underwent content validation among five patients with hypertension. Five patients were selected based on previous literature proposing the minimum number of acceptable content experts as two to six.^18^ Patients who were aged 40–70 years, were diagnosed with essential hypertension (secondary causes ruled out), were on anti-hypertensive treatment and had a good understanding of the Malay language were included. The content validation process was conducted as a face-to-face meeting. Patients were recruited from the primary care clinic, shown a PowerPoint presentation of the storyboards of the videos and asked to rate the relevance of the contents. It was essential for patients to review the VBIs designed to assess their comprehension and understanding of the videos’ storyboard and script. The feedback gained from this session guided further modifications made to the VBI storyboard or script.

At this stage, two main actors were identified for the production phase. Actor 1 would be the main host throughout the five videos, while Actor 2 would play the role of a patient with hypertension. Both these actors voluntarily participated; thus, no payment was involved.

### Phase 2: Production

This phase involved the recording of the VBI series. The video recording was conducted at the university primary care clinic using a Xiaomi 11T handphone model with MIUI version 14.0.4. (8GB + 256GB, LPDDR4X RAM + UFS 3.1 storage manufactured by Xiaomi Inc. Haidian District, Beijing China). Narration on the videos was recorded by Actor 1 using a Rode Lavalier GO Omnidirectional Lavalier Microphone For Wireless GO Systems (3.5mm TRS input & output manufactured in Sydney, Australia) microphone. For each video, several attempts of recording were made until the research team was satisfied with the video recordings.

### Phase 3: Post-production

This phase involved video editing and face validation among patients with hypertension. The videos were edited using CapCut Pro version 2.1.0-beta 4 by the main researcher with the assistance of an IT expert. The editing focused on the insertion of animation, video sequences and sound quality or effect. After the videos were edited, the research team reviewed them prior to face validation. The total cost covered the editing services from the professional IT expert, which was approximately RM 1000 per video. The production phase did not involve any costs, as it was conducted by the research team using existing recording equipment.

Face validation involved 10 patients with hypertension. These patients were asked to assess the clarity, comprehension, video quality, persuasive content and character identification of the VBI series. Some authors recommend 6 to 20 participants for technology or instrument validation.^20^ In this study, face validation was conducted using the item-level face validity index (I-FVI), in which clarity and comprehension were scored from 1 (‘not clear and not understandable’) to 4 (‘very clear and understandable’). Items scoring 1 or 2 were re-categorised into the ‘not clear and not understandable’ category with 0 points each. Items scoring 3 or 4 were re-categorised into the ‘very clear and understandable’ category with 1 point each. The acceptable cut-off I-FVI should be at least 0.80.^21^ Depending on the feedback obtained from patients during face validation, the videos were modified when needed.

## Results

### Phase 1: Pre-production

For the first round of content validation, four video storyboards (Videos 2, 3, 4 and 5) achieved an I-CVI of 1.0. Video 1 (balanced diet for patients with high blood pressure) achieved an I-CVI of 0.67 only. This was due to the lack of specific dietary information and low-salt diet recommendations for patients with hypertension. The I-CVI from the first round of content validation is summarised in Table 3. Revisions were made to the storyboard of Video 1 by adding examples of common Malaysian meals using the concepts of Healthy Plate and DASH. Recommended daily salt intake/portion based on Medical Nutrition Therapy guidelines was also added. An I-CVI of 1.0 was achieved for Video 1 after the second round of content validation.

**Table 3:** I-CVI among the content expert panel for the first round of content validation.

Video title	Content expert 1 (FMS)	Content expert 2 (FMS)	Content expert 3 (Cardiologist)	Content expert 4 (Pharmacist)	Content expert 5 (Dietician)	Content expert 6 (Physiotherapist)	I-CVI
1. Balanced diet for patients with high blood pressure	1	0	1	1	0	1	0.67
2. Weight loss and physical activity for patients with high blood pressure	1	1	1	1	1	1	1.0
3. Cessation of smoking and reduction of alcohol consumption	1	1	1	1	1	1	1.0
4. Method of taking a blood pressure reading at home	1	1	1	1	1	1	1.0
5. Adherence to medication for high blood pressure	1	1	1	1	1	1	1.0

FMS, family medicine specialist; I-CVI, item-level content validity index

Five patients with hypertension who fulfilled the inclusion criteria were conveniently recruited from the primary care clinic. All five videos attained an I-CVI of 1.0 in terms of relevance of contents. Most patients suggested inserting animations into the videos to make them interesting.

### Phase 2: Production

The five videos were recorded following the storyboard and script, which achieved agreement during content validation. All videos had an acceptable quality with a dimension of 1920x1080 pixels (high definition). An introductory video, which lasted 2 min 34 s, was also added to the series as an introduction to the objectives of the VBI series for patients. It highlighted the definition of hypertension, its risk factors and how self-care practices play an important role in the management of blood pressure control. This step aimed to spark interest for patients to watch all five subsequent videos as part of their hypertension management at home.

### Phase 3: Post-production

Recording lasted an average of 48 h, while editing of each video lasted 4 days. The length of all recorded videos ranged from 5 min 10 s to 6 min 42 s. Table 4 shows the summary and duration of each video in the VBI series.

**Table 4:** Summary and duration of the video-based intervention series.

Video	Summary	Duration
*Video pengenalan kepada siri ini* (Introduction video to this series) 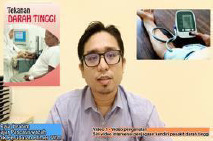	Introduction to hypertension, its risk factors and self-care practices among patients with hypertension to control blood pressure	2 min 34 s
**VIDEO 1** *Pemakanan seimbang untuk pesakit darah tinggi* (Balanced diet for patients with high blood) 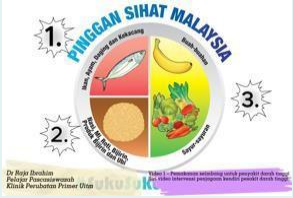	To improve self-care practice through dietary changes based on the DASH diet and Healthy Plate guideline	6 min 24 s
**VIDEO 2** *Penurunan berat badan dan aktiviti fizikal untuk tekanan darah tinggi* (Weight loss and physical activity for patients with high blood pressure) 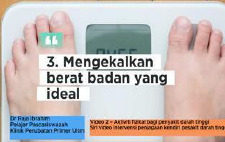	To improve self-care practice through physical activity and weight loss	5 min 10 s
**VIDEO 3** *Berhenti merokok dan pengambilan alcohol* (Cessation of smoking and reduction of alcohol consumption) 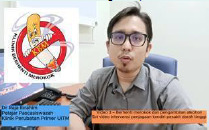	To improve self-care practice by quitting smoking and reducing alcohol consumption	6 min 40 s
**VIDEO 4** *Cara mengambil bacaan tekanan darah di rumah* (Method of taking a blood pressure reading at home) 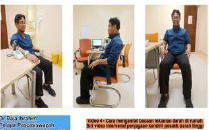	To improve self-care practice through self-monitoring of blood pressure, with the steps of measuring blood pressure explained	6 min 42 s
**VIDEO 5** *Pematuhan kepada ubat untuk tekanan darah tinggi* (Adherence to medication for high blood pressure) 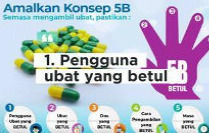	To improve self-care practice through adherence to medication by highlighting the 5B concept	6 min 13 s

DASH, dietary approach to stop hypertension

Face validation was conducted on 10 patients with hypertension who fulfilled the same inclusion criteria used in content validation. The I-FVI calculated for all five videos was 1.0, as shown in Table 5. The patients perceived that the videos were of good quality; they agreed that there was persuasive content and that they were able to identify with the characters.

**Table 5:** I-FVI among 10 patients with hypertension.

Video title	Patient 1	Patient 2	Patient 3	Patient 4	Patient 5	Patient 6	Patient 7	Patient 8	Patient 9	Patient 10	I-FVI
1. Balanced diet for patients with high blood pressure	1	1	1	1	1	1	1	1	1	1	1.0
2. Weight loss and physical activity for patients with high blood pressure	1	1	1	1	1	1	1	1	1	1	1.0
3. Cessation of smoking and reduction of alcohol consumption	1	1	1	1	1	1	1	1	1	1	1.0
4. Method of taking a blood pressure reading at home	1	1	1	1	1	1	1	1	1	1	1.0
5. Adherence to medication for high blood pressure	1	1	1	1	1	1	1	1	1	1	1.0

## Discussion

In this study, a VBI series of five videos on self-care practices for patients with hypertension was developed and validated. It underwent robust validation processes, which included content validation by both panel experts and patients as well as face validation by patients. To our knowledge, there are educational videos in the Malay language available for patients with hypertension that can be obtained from YouTube and Ministry of Health websites.^22^ However, the development and validation of these videos are not published. Recently, Hamid et al.^23^ developed and validated the contents of a video on dietary adjustments among Malaysian patients with hypertension, but the video is still in production.

The content validation of the VBI series maintained methodological rigour, in which the invited expert panel had the knowledge, skills and experience in managing and treating patients with hypertension in clinical practice. The expert panel was able to discuss and assess the relevance of the themes and contents presented in the storyboards and scripts. In this way, they identified the important key areas of each video and provided valuable feedback for the production of educational material relevant to the self-care practices of patients with hypertension.

During the first round of content validation, there were some concerns in Video 1 about the lack of information presented in the storyboard regarding low-salt diet recommendations and examples of common Malaysian meals that would fulfil the DASH diet and Healthy Plate guidelines for patients with hypertension. The expert panel suggested that the researchers include meals that local Malaysian patients could apply in their daily meals rather than Western meals. These recommendations are in line with reported barriers and facilitators towards hypertension-friendly diets. Patients find it difficult to control their choice of meals due to the widespread availability of food in Malaysia, including home-cooked meals that are high in salt.^24^ Thus, the inclusion of these recommendations would aid patients and their family members in following the recommended diet.

This study conducted content validation of the video storyboards and scripts among the intended users of the VBI series – patients with hypertension. Studies have shown that educational materials should be validated by the target audience to which the material is intended because they will be the ones making use of the technological tools.^25^ In their study, Do Nascimento et al. developed an educational video to promote self-efficacy in preventing childhood diarrhoea, which was validated among 17 mothers whose children were under 5 years old.^26^ The video was found to be valid and reliable, reflecting the importance of including the intended target population of the developed materials during the development and validation processes.

The final duration of the five videos in the VBI series ranged from 5 min 10 s to 6 min 42 s. According to Guo et al.,^27^ the median engagement time for videos less than 6 min long is close to 100% and should be reduced to 50% and subsequently to 20% with an extension from 12 min to 40 min. Shorter videos under 5 min should concentrate on particular subjects and take only a minimal amount of time to watch, making them more effective in grabbing learners’ interest and attention.^28^ Conversely, Kim et al.^29^ found that patients were able to tolerate videos that were more than 3 min. In our study, we found it difficult to create videos of shorter duration, as a significant amount of information needed to be shared to be able to motivate patients to make changes to their self-care practices.

Video assessment by the target audience after the final development phase is crucial to ensure that the video developed presents the intended content. In this study, face validation was conducted, yielding a satisfactory I-FVI; the value indicated that the VBI series was clear and easy to comprehend. In a previous study, a newly developed nutrition resource kit with an educational video was face-validated by 12 at- risk or malnourished older adults. The process revealed excellent face validity.^30^

### Study strengths and limitations

The strength of this study is that the VBI series underwent a rigorous process of development and validation involving an expert panel and patients with hypertension. In contrast, the limitation is that the study was conducted at only one centre with mainly Malay patients, which limits the generalisation of the results. Another limitation is that the video series was developed and edited by the main researcher, who had a medical background with limited IT skills. Moreover, there was a lack of a Malay language-certified expert involved in the development phase to ensure the precision and appropriateness of the terminologies and phrases in the Malay language used in the VBI series. The research team members were native speakers of the Malay language; hence, the terminologies and phrases used within the instrument were pitched at the basic and intermediate levels of the language instead of the high level.

### Recommendations for future research and clinical practice

Future research should consider involving multi-centred primary care clinics and developing a VBI series in the Mandarin or Tamil language to ensure adequate representation of the main ethnicities in Malaysia. The development of a VBI series should involve professional IT experts and videographers to produce high-quality videos with persuasive content. Lastly, a randomised control trial should be conducted to evaluate the effect of the VBI series on the self-care practices of patients with hypertension aged 18-80 years.

## Conclusion

In this study, five validated videos were developed, which attained acceptable content and face validation indices among the expert panel and patients with hypertension. This VBI series can be useful in promoting selfcare practices, but further studies should be conducted to determine its effectiveness on patients with hypertension in primary care settings.

## Data sharing statement

Raw data are available upon request with permission from the corresponding author.

**How does this paper make a difference in general practice?**

- Video-based interventions (VBIs) are increasingly being used in health promotion. They have the advantages of being sustainable and accessible, with patients being able to play them repeatedly from the convenience of their homes. Thus, the validated VBI series developed in this study is useful in promoting self-care practices among patients with hypertension in primary care settings.
